# Effects of local versus global competition on reproductive skew and sex differences in social dominance behaviour

**DOI:** 10.1098/rspb.2022.2081

**Published:** 2022-11-30

**Authors:** Olof Leimar, Redouan Bshary

**Affiliations:** ^1^ Department of Zoology, Stockholm University, 106 91 Stockholm, Sweden; ^2^ Institute of Biology, University of Neuchâtel, Neuchâtel, Switzerland

**Keywords:** social hierarchy, mating interference, foraging interference, aggression, hard and soft selection, game theory in biology

## Abstract

Social hierarchies are often found in group-living animals. The hierarchy position can influence reproductive success (RS), with a skew towards high-ranking individuals. The amount of aggression in social dominance varies greatly, both between species and between males and females within species. Using game theory we study this variation by taking into account the degree to which reproductive competition in a social group is mainly local to the group, emphasizing within-group relative RS, or global to a larger population, emphasizing an individual’s absolute RS. Our model is similar to recent approaches in that reinforcement learning is used as a behavioural mechanism allowing social-hierarchy formation. We test two hypotheses. The first is that local competition should favour the evolution of mating or foraging interference, and thus of reproductive skew. Second, decreases in reproductive output caused by an individual’s accumulated fighting damage, such as reduced parenting ability, will favour less intense aggression but should have little influence on reproductive skew. From individual-based simulations of the evolution of social dominance and interference, we find support for both hypotheses. We discuss to what extent our results can explain observed sex differences in reproductive skew and social dominance behaviour.

## 1. Introduction

In group-living animals, positions in a social hierarchy are often established and maintained through pairwise aggressive interactions. The intensity of this aggression varies greatly, both between species and between males and females within species, with females typically showing less aggression than males [1–3]. There is also variation in the magnitude of reproductive skew caused by social dominance [3–5]. A traditional explanation for sex differences in reproductive skew and dominance behaviour is that there is greater scope for male than for female variation in reproductive success (RS), sometimes referred to as Bateman’s principle [1–3,6].

We analyse the evolution of reproductive skew and dominance behaviour by investigating the range from local to global competition in a metapopulation of local groups that do not interact aggressively. The reasoning can apply either to males or to females. For our analysis, effects of local versus global competition on contested RS is the thing that matters. There is purely local competition if the group reproductive output is independent of reproductive skew, in which case a top-ranked individual in principle should have the capacity to make up a full group output. This individual would then have an incentive to monopolize reproduction in the group. For purely global competition, an individual’s RS should instead be measured against those in the larger population, so that there is little or no incentive to interfere with the reproductive output of other group members. The terms ‘soft selection’ and ‘hard selection’ are sometimes used for such a distinction between local and global competition [7]. This terminology for the scale of competition comes from work on interactions between relatives (e.g. [8]) but, as we do here, it can also be applied to interactions between non-relatives [9]. For the evolution of reproductive skew and dominance behaviour, an important difference between local and global competition might then be that local competition favours mating or foraging interference (henceforth, interference). Interference can increase an individual’s relative RS in a group, which is favoured by local competition, whereas global competition only favours a high absolute RS. Concerning the evolution of sex differences in reproductive skew and dominance behaviour, evolution in males might be closer to local competition and in females to global competition, although with many intermediates between the extremes.

Apart from the scale of competition, various reproductive consequences of contest damage are likely to influence the evolution of dominance behaviour. Mortality from contest damage can decrease or eliminate reproduction and is one such effect. A reduced phenotypic quality that lowers parenting success could be a more widespread example, in particular in females [1–3]. Our aim is to elucidate the combined influence of the scale of competition (from local to global) and the costs of contest damage (in particular reduced parenting ability) on the evolution of dominance behaviour, interference and reproductive skew. Our hypotheses are that interference is favoured by local competition and has a strong influence on the evolution of reproductive skew, and that other reproductive consequences of contest damage will influence the intensity of aggression, but will have a weaker effect on reproductive skew. As an alternative, we also consider situations where interference is very costly for dominants to perform and/or less effective in reducing subordinate reproduction, which should lead to less skew and less fighting. By investigating these hypotheses we aim to explain important aspects of sex and species differences in reproductive skew and dominance behaviour. How local versus global competition might influence the evolution of interference in social dominance has not previously been studied using game theory.

Our game-theory model is similar to previous approaches in using learning about differences in fighting ability as an evolving behavioural mechanism that can give rise to within-sex dominance hierarchies [10–12]. This kind of model of the gaining of information in contests qulitatively resembles the sequential assessment game [10,13], and similar behavioural mechanisms have previously been used to model social dominance [14].

Our current model includes mating or foraging interference by dominants as a new element that has not previously been studied in a model. This is done by introducing the strength of interference by dominants in subordinate reproduction as a separate trait that can evolve. The effect of variation in this trait spans from no interference to dominants nearly eliminating subordinate reproduction. We conceptualize dominance interactions in a group as divided into two phases. A dominance hierarchy is first established, and we assume that the hierarchy imposes a baseline level of reproductive skew, arising from such things as differences in the qualities of display arenas on a lek or of breeding sites, or possibly female preferences for males of different ranks. Interference can then increase reproductive skew above this baseline. Furthermore, in our previous model [12] we assumed that competition was purely local, meaning that dominance interactions did not influence the total group reproductive output (e.g. males contributing matings but females determining the reproductive output). In the current model, we examine different degrees of local competition, from purely global to purely local competition. By purely global competition we mean that each individual’s RS is a function of the amount of resources that individual acquires (in some species females can be close to global competition). Finally, we examine the influence of the amount of uncontested RS that individuals can achieve outside of the current dominance interactions, varying from a small amount (which we focus on in the main text) to a substantial amount. Our previous model [12] also examined outside-option RS.

In the following we outline the model elements and present results from individual-based evolutionary simulations. The genetically determined traits in the model are the components of a reinforcement learning [15] mechanism, as used previously [11,12], together with the strength of interference. We examine the evolution of these traits in one of the sexes, which could be either males or females. We then discuss to what extent our results provide a qualitative explanation of between-sex differences, and also if the factors we identify can throw light on within-sex species differences in reproductive skew and dominance behaviour. Finally, our analysis uses game theory to address the general question of why there is variation in the intensity of aggression, which was raised by Maynard Smith & Price [16] in their seminal contribution to game theory in biology, and we end by discussing sex differences in dominance behaviour from this perspective.

## 2. The model

Our model here is an extension of previous models [11,12], by adding variation in the degree of local competition and introducing interference as a trait. In previous models, competition was local, in the sense that dominance behaviour did not influence the total group reproductive output, but there was no interference, such that an individual’s RS was assumed to be directly determined by the dominance position it achieved. This means that in previous models, the amount of reproductive skew from social dominance was a model assumption and not a consequence of trait evolution. Here, we introduce interference as a separate trait that influences an individual’s relative RS and study the co-evolution of this trait with other traits that determine the formation of a social hierarchy. Previously, we examined two types of costs of fighting damage, a decrease in the effective fighting ability and a risk of mortality from damage [12]. Here, we add another cost of fighting, viz. a decreased parenting ability from fighting damage.

**Figure 1.**
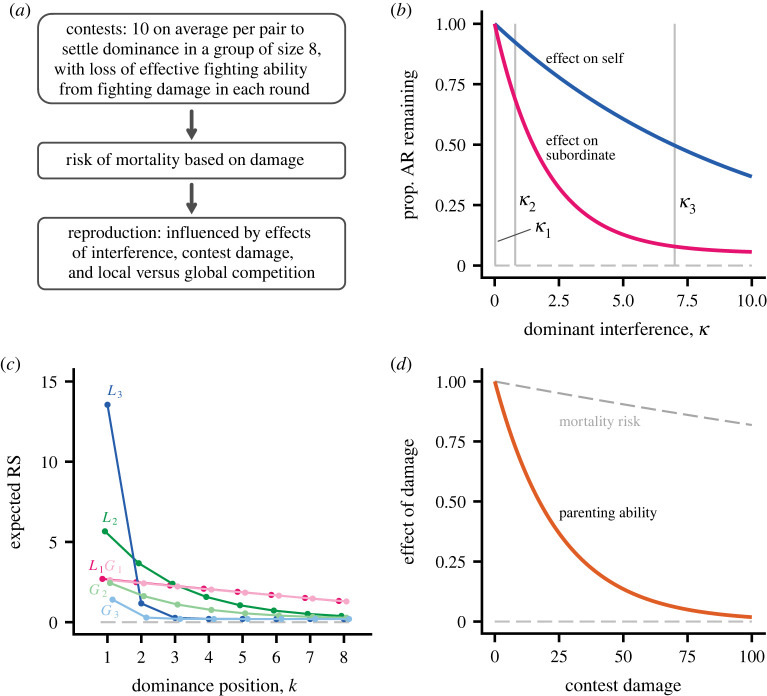
Illustration of model elements, with a one-season life cycle. The focus is on one of the sexes, either males or females. (*a*) Dominance-hierarchy formation comes first, followed by risk of mortality, which depends on contest damage, and reproduction. An individual’s reproductive success (RS) is the net result of different effects. (*b*) After hierarchy formation, dominant individuals can interfere with subordinates, reducing their acquired resources (AR), for instance matings for males or foraging opportunities for females. Interference strength is a genetically determined trait *κ*. The red curve shows the decrease in AR for a subordinate and the blue curve shows the decrease for a dominant from performing interference. The grey vertical lines indicate three values of *κ*. (*c*) Expected RS as a function of dominance position (*k* = 1 is top ranked) for local (*L*_1_, *L*_2_, *L*_3_) and global (*G*_1_, *G*_2_, *G*_3_) competition, corresponding to trait values *κ*_1_, *κ*_2_, *κ*_3_ from (*b*). The curves do not include effects of contest damage. Other local groups are assumed to have a total RS of 16 offspring. (*d*) The orange curve shows the influence of accumulated contest damage on parenting ability and the sloping dashed curve shows the risk of mortality from contest damage. (Online version in colour.)

The elements of our model are outlined in figure 1. First a hierarchy is formed through aggressive interactions [17], then there is a risk of mortality from fighting damage, followed by reproduction and interference. Interference is a trait (*κ*) that measures how strongly an individual of a given dominance rank acts to reduce the reproduction of those of lower rank. Interference causes a proportional reduction in acquired resources (AR), i.e. the contested resources for reproduction acquired by a subordinate (figure 1*b*). For males, AR will include the basic capacity to deliver matings (which for instance might differ between display arenas), but could also include foraging and other components of paternal care. For females, AR might consist of foraging opportunities. Mating and foraging interference decrease an individual’s AR. Interference is costly to perform (figure 1*b*), and we assume that effects caused by different individuals interact multiplicatively. For both local and global competition, interference has the effect of increasing reproductive skew, but for global competition there is the additional effect of reducing the total group reproductive output (figure 1*c*). In addition to interference, we assume that accumulated contest damage can cause a proportional reduction in the individual’s parenting ability (figure 1*d*).

To avoid the possibility that strong interference causes some individuals to be entirely without reproductive prospects, we assume that there is a small probability of ‘outside-option’ reproduction. The effect of this can be seen in figure 1*c*, where the curves labelled *L*_3_ and *G*_3_ come fairly close to, but do not reach zero for the bottom dominance positions (high values of *k*).

Many of the details of the model are the same as in previous models [11,12], in particular the traits of the reinforcement-learning mechanism, but for completeness a full description is given in electronic supplementary material, including a table of notation and definitions for the model (electronic supplementary material, table S1).

### (a) Evolutionary simulations

Individuals are assumed to have genetically determined traits. The evolution of the traits is studied in individual-based simulations (table 1). The traits for individual *i* include the strength of interference, *κ*_*i*_, and a number of traits of the reinforcement-learning mechanism. Of these, the degree of generalization, *f*_*i*_, expresses how strongly an individual generalizes learning about one opponent to other opponents, which is important for winner–loser effects. There are also the preference and value learning rates, *α*_*θi*_, *α*_*wi*_; the bystander learning rate, *β*_*i*_; the initial preference for the aggressive action A, *θ*_0*i*_; and the initial estimated value of a round, *w*_0*i*_. These are basic reinforcement-learning traits. Finally, the effect of observations on preference and value functions, *γ*_0*i*_, *g*_0*i*_, and the perceived reward from performing A, *v*_*i*_, are assumed to be genetically determined traits. See electronic supplementary material, table S1 and text for further explanation.

**Table 1.** Trait values (mean ± s.d. over 100 simulations, each over 5000 generations) for six different cases of individual-based evolutionary simulations of social dominance interactions. The parameters that vary between cases are proportion local versus global competition (*λ*) and the cost of contest damage (*c*_2_).

case	*κ*_*i*_	*f*_*i*_	*α*_*θi*_	*α*_*wi*_
1 : *λ* = 0.0, *c*_2_ = 0.00	0.020 ± 0.005	0.079 ± 0.017	52.2 ± 6.7	0.031 ± 0.005
2 : *λ* = 0.5, *c*_2_ = 0.00	0.799 ± 0.160	0.089 ± 0.019	54.5 ± 7.6	0.032 ± 0.005
3 : *λ* = 1.0, *c*_2_ = 0.00	6.759 ± 0.330	0.253 ± 0.022	22.5 ± 6.1	0.032 ± 0.011
4 : *λ* = 0.0, *c*_2_ = 0.04	0.020 ± 0.004	0.096 ± 0.011	134.8 ± 17.1	0.116 ± 0.015
5 : *λ* = 0.5, *c*_2_ = 0.04	0.603 ± 0.147	0.091 ± 0.010	124.9 ± 14.3	0.058 ± 0.012
6 : *λ* = 1.0, *c*_2_ = 0.04	6.510 ± 0.423	0.254 ± 0.025	52.1 ± 10.5	0.026 ± 0.008

case	*β*_*i*_	*θ*_0*i*_	*w*_0*i*_	*γ*_0*i*_	*g*_0*i*_	*v*_*i*_
1	1.10 ± 0.18	4.77 ± 0.19	−0.07 ± 0.01	1.20 ± 0.27	0.07 ± 0.01	0.82 ± 0.01
2	0.51 ± 0.14	4.90 ± 0.15	−0.02 ± 0.01	0.62 ± 0.17	0.04 ± 0.01	0.93 ± 0.01
3	0.18 ± 0.14	5.52 ± 0.38	0.01 ± 0.03	0.68 ± 0.41	0.05 ± 0.02	0.95 ± 0.01
4	2.33 ± 0.18	4.12 ± 0.16	−0.19 ± 0.02	2.50 ± 0.22	0.06 ± 0.01	0.57 ± 0.02
5	1.02 ± 0.18	5.14 ± 0.22	−0.08 ± 0.02	1.74 ± 0.24	0.06 ± 0.01	0.74 ± 0.03
6	0.18 ± 0.11	5.08 ± 0.28	−0.02 ± 0.01	0.61 ± 0.29	0.04 ± 0.01	0.94 ± 0.01

In evolutionary simulations, each trait is determined by an unlinked diploid locus with additive alleles. Alleles mutate with a probability of 0.002 per generation, with normally distributed mutational increments. The standard deviation of mutational increments for each trait was adjusted to ensure that simulations could locate evolutionary equilibria (as seen in table 1, the evolved traits vary in scale, and mutational increments need to reflect the scale of trait variation).

A simulated population consisted of 500 groups of eight individuals taking part in dominance interactions (either males or females), plus eight individuals of the other sex, resulting in a total population size of *N* = 8000. Each interacting individual was assigned a (non-genetic) quality *q*_*i*_, independently drawn from a normal distribution with mean zero and standard deviation *σ*_*q*_. As a simplification we assume that all offspring disperse globally over all groups, to form the adults of the next generation. For each case reported in table 1, simulations were performed over intervals of 5000 generations, repeated at least 100 times, to estimate mean and standard deviation of traits at an evolutionary equilibrium.

#### (i) Standard parameter values

The following ‘standard values’ of parameters (electronic supplementary material, table S1) were used: proportion of local competition, *λ* = 0.0, *λ* = 0.5 or *λ* = 1.0; probability of an offspring being produced through uncontested, outside-option reproduction, *Q* = 0.1; distribution of individual quality, *σ*_*q*_ = 0.50; damage cost parameters, *c*_0_ = 0.02, *c*_1_ = 0.0004, *c*_2_ = 0.00 or *c*_2_ = 0.04; interference parameters *b*_0_ = 0.1, *b*_1_ = 0.5, *ϕ* = 0.95; observations of relative quality, *a*_0_ = 0.707, *σ* = 0.50; perceived penalty variation, *σ*_*p*_ = 0.25. For these parameters, around 50% of the variation in the observations by individuals in a round is due to variation in relative fighting ability, *q*_*i*_ − *q*_*j*_.

## 3. Results

The trait values that evolved in our individual-based simulations are shown in table 1, for different degrees of local competition and absence versus presence of a cost of decreased parenting ability from damage. The strength of interference (*κ*_*i*_) for the three different degrees of local competition (*λ* = 0.0, 0.5, 1.0) correspond approximately to the three values illustrated in figure 1*b*, with greater interference for higher degree of local competition (table 1). In contrast, the evolved interference traits were similar for absence versus presence of a cost of decreased parenting ability (*c*_2_ = 0.00 versus *c*_2_ = 0.04, table 1). These results are predicted by our hypotheses.

**Figure 2.**
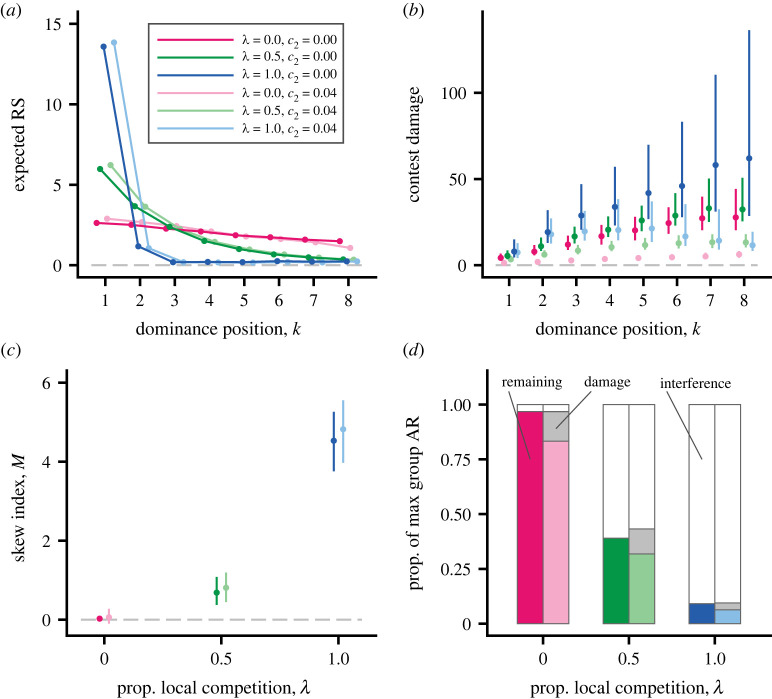
The outcome of individual-based simulations, showing effects of local versus global competition and contest damage. (*a*) Expected RS as a function of dominance position for the six cases in table 1 (colour coding given in legend). Red, green and blue show global, intermediate and local competition, respectively, with risk of mortality from figure 1*d*. The light-coloured curves in addition have reduced parenting ability from contest damage, as given in figure 1*d*. (*b*) Accumulated contest damage as a function of dominance position, for the six colour-coded cases in (*a*). Points and bars give median and first and third quartiles for the distribution over simulated local groups. (*c*) Median and first and third quartiles for the distribution over groups of the skew index *M* [18] (see electronic supplementary material, table S1), as a function of the proportion of local competition (the six colour-coded cases are shown). (*d*) Effects of interference and contest damage on the total group contested AR (summed over competing individuals), expressed as proportions of the maximal possible value. Unfilled parts indicate costs of interference, light grey parts costs of damage (reduced parenting ability), and colour-coded parts the remaining AR, respectively. (*a,b*) Include only surviving individuals; these have a dominance position. There were 500 groups in the simulated populations. (Online version in colour.)

Figure 2 shows different aspects of the outcome of dominance and interference interactions for the cases in table 1. The degree of local competition (*λ*) had a strong effect on the distribution of RS over ranks (figure 2*a*) and thus on reproductive skew (figure 2*c*), with higher skew when competition is more local, whereas the absence versus presence of a cost of decreased parenting ability only weakly influenced these measures (figure 2*a*,*c*). In contrast, both more local competition and the absence of a parenting-ability cost of damage lead to higher contest damage (figure 2*b*). In addition, contest damage tended to be higher for lower-ranked individuals, in particular without a cost of decreased parenting ability (figure 2*b*).

A comparison of the total group AR contributed by the competing sex for the different cases in table 1 appear in figure 2*d*. In the cases with full local competition (*λ* = 1), interference strongly decreased the group AR. To interpret this, one can note that for full local competition, members of the competing sex (e.g. males) only contribute matings, but no additional resources to offspring. These instead come from the other sex (e.g. females). The sharp decrease in AR with full local competition thus only means that interference prevents most members of the competing sex from achieving contested matings. The interpretation of the cases with intermediate degree of local competition (*λ* = 0.5), and intermediate strength of interference, could instead be that a substantial part of the AR contributed by the competing sex is not subject to interference (e.g. nesting sites), but that interference can exclude individuals from other substantial parts (e.g. foraging areas). With global competition (*λ* = 0.0), there is very little interference and the only noticeable decrease in group AR comes from a small reduction in parenting ability from fighting damage (figure 1*d*).

**Figure 3.**
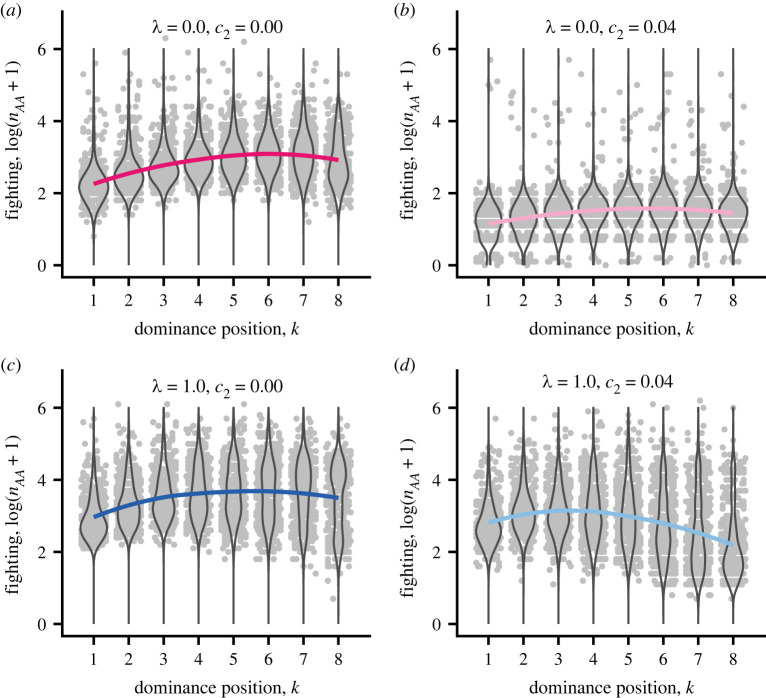
Simulated data (light grey points), density violins and colour-coded fitted curves of log-transformed total number of AA rounds (fighting rounds, loess fits) for cases with either no or full local competition. (*a–d*) Cases 1, 3, 4 and 6 in table 1, with colour coding as in figure 2. The *x*-values of the points are jittered for visibility. (Online version in colour.)

The amount of fighting for the different ranks, and for local versus global competition and absence/presence of a parenting-ability cost of damage is shown in figure 3, with examples of single groups in electronic supplementary material, figure S1. There is much variation in the number of fighting rounds between individuals in different groups, but the tendency is that intermediate ranks fight the most. The tendency for the lowest ranks to fight less is an example of an ‘opt-out loser effect’, which we studied previously [12]. Part of the variation in fighting is that some pairs of individuals did not fight at all (electronic supplementary material, figure S2). This was most common for global competition with a parenting-ability cost, and tended to occur when one of the individuals had low rank and the opponent had a higher rank (electronic supplementary material, figure S2).

The overall amount of fighting also varied substantially between the cases, being around 10 times lower for global competition with a parenting-ability cost than for local competition without parenting-ability cost (figure 3*b* versus *c*).

We also investigated the evolutionary consequences of a substantially higher probability of outside-option reproduction (*Q* = 0.5, electronic supplementary material, table S2 and figures S3–S5). This did not strongly influence interference (see *κ*_*i*_ in table 1 and electronic supplementary material, table S2), but changed the learning traits in such a way that fights became shorter and less damaging (electronic supplementary material, figures S3c, S4 and S5). Because there was more outside-option reproduction, reproductive skew was reduced (electronic supplementary material, figure S3a,c). With absence of a parenting ability cost, the lower ranks still tended to accumulate higher damage than the top ranks (electronic supplementary material, figure S3b), but with full local competition and decreased parenting ability from damage the lowest ranks showed noticeably less fighting than the top ranks (electronic supplementary material, figure S4d).

Finally, we examined the evolutionary consequences of a substantially more costly and less effective interference. As an example, for full local competition (*λ* = 1.0) and changing the interference parameters such that the lower curve in figure 1*b* gives the effect on self and the upper curve the effect on a subordinate, the outcome was that the interference traits evolved to near zero, leading to a baseline reproductive skew, and less fighting damage (electronic supplementary material, figure S6).

## 4. Discussion

Our evolutionary analysis showed that more intense local (within-group) competition favours stronger mating and/or foraging interference by dominants (figure 2*d*, table 1), reducing the RS of subordinates and increasing the reproductive skew (figure 2*a*,*c*). We also found that costs in the form of reduced parenting ability from contest damage can sharply reduce fighting and the damage from fighting (figures 2*b* and 3). These factors, separately or acting together, have effects that are large enough to potentially explain observed sex differences in social dominance behaviour. We further examined how fighting and damage varied between dominance positions, finding that individuals of intermediate or low ranks fought most and suffered the most contest damage (figures 2*b* and 3).

The results on interference were achieved by introducing an evolving interference trait into the model. This is a new element compared to previous models of social-hierarchy formation that are similar to our current model in using reinforcement learning as a behavioural mechanism [11,12]. Interference might correspond to different types of behaviours, ranging from dominant males attacking and chasing subordinate males to prevent them from mating, to dominant females excluding subordinate females from foraging areas. Among the examples are males of Alpine ibex, for which a dominance hierarchy is established before the start of the mating season [19,20], and dominant, lactating olive baboon females excluding subordinate females from foraging through aggression [21].

Interference, as used in our model, is related to punishment in animal societies, for which social dominance is a major example [22]. One idea is that punishment serves to deter cheating and promote cooperation, and another, contrasting idea is that it serves to change the relative RS in a group [9,23]. In our model, interference plays the latter role, and can be costly by reducing the AR of the interfering individual. Interference resembles the concept of spite as used in theoretical studies on kin selection, with the conclusion that local competition favours spite [8]. Nevertheless, because we assume interacting individuals to be unrelated, interference in our model is not spite in the kin-selection sense [24].

In our model there are two contributions to reproductive skew: the ‘starting’ distribution of AR over dominance ranks, which is the distribution for zero interference (*κ* = 0), and the change from this due to interference by dominants. Either of these contributions can vary between situations, giving rise to many possibilities. In addition, the cost and effectiveness of interference can vary, and if the cost becomes too high or effectiveness too low, interference will not evolve (see electronic supplementary material, figure S6). This could, for instance, correspond to situations where the synchrony of receptivity of females in a group makes it difficult or infeasible for high-ranking males to monopolize matings, as has been found in primate species [25].

To determine whether our model results explain observed sex differences in social dominance behaviour, one would need data on the scales of male and female competition in different species, as well as data on the influence of fighting damage on parenting ability, or similar effects of disturbance from fighting. Although these possible explanations have been put forward [1,2], there seems to be a lack of quantitative estimates. Still, studies on female-female competition through interference in social groups support the general idea that interference is stronger when it can increase the RS of a dominant individual [21,26–28].

Concerning the scale of competition, so-called female reproductive dominance is often assumed in life-history modelling (e.g. [29]). In species living in social groups, this would imply that male-male competition is mainly local, but there seem to be no studies directly investigating it. The concepts of hard and soft selection (the terminology is from [30]) are much used in studies of metapopulations [7] and correspond to global versus local competition, but again there is little in the form of empirical estimates of these forms of population regulation. In the context of population management and conservation, data on culling, sterilization or harvesting of either males or females are often studied (e.g. [31–33]), which in principle could allow estimates of the scale of competition, but up to now such data have not been used for this purpose.

One way to assess costs of dominance interactions on parenting ability is to examine genetic correlations between the corresponding traits, because such correlations could indicate an evolutionary trade-off. Evidence for such a trade-off has been found in female baboons [34], with higher risk of miscarriage and sometimes reduced fertility in high-ranking females, and in cows [35], with lower fertility and milk production in individuals more adapted for fighting. This complements the many observations consistent with decreased parenting ability from fighting [1,2].

Hormonal manipulation is another way to examine such a trade-off, and this has been performed on cleaner fish in the wild [36]. Cleaner fish (*Labroides dimidiatus*) live on coral reefs. They forage by removing ectoparasites from other fish, so-called clients [37], and are organized into dominance hierarchies of females and a top-ranking male that has undergone sex reversal [38]. The study [36] found that testosterone-injected females increased their aggression towards subordinate females and spent less time interacting with clients, which supports our model assumptions. In general, cleaner fish, could be an example where female competition is relatively close to global, because they forage on non-monopolizable resources (clients) and reproduce through pelagic eggs.

There are studies on injuries and wound healing in baboons that support our results that high ranks suffer less damage, both in males [39] and in females [40]. There is also qualitative support that health and longevity is higher for high-ranking individuals in many social mammals, with most relations being observed for females [41]. It has been argued [42] that improved health and longevity for high ranks typically applies to females, whereas high-ranking males might suffer greater costs. This conclusion is based on measurements of stress hormones. It is uncertain to what extent this holds for other measures of costs, as it was found that high-ranking male baboons, with high concentrations of stress hormones, still had faster wound healing [39]. It seems that the general topic would profit from further empirical studies.

Concerning sex differences in contest damage, there are data for primate species showing that males receive more injuries than females [43]. Most likely, similar results could be found in many social species.

There are a number of previous models of reproductive skew, which have a focus on cooperative breeding among related individuals [44–50], but these models could also apply to the situations we study here. To illustrate the similarities and differences between our model and these previous reproductive-skew models, we briefly compare with one of these [50]. It is a further development of so-called ‘tug-of-war’ models, modified to better correspond to the situation for males in many primate species. One modification [50] from previous ‘tug-of-war’ models is to assume local competition. In our model, we also examine the case of pure local competition (*λ* = 1). An important prediction from the model [50] is that there are lower costs of aggression when a greater proportion of RS is uncontested (e.g. because of female reproductive synchrony), and our model agrees with this (compare figure 2*b*, for which *Q* = 0.1, with electronic supplementary material, figure S3b, for which *Q* = 0.5). Another prediction [50] is that dominants invest more into conflicts than subordinates. This appears to differ from our model, in the sense that we find that top-ranked individuals suffer less contest damage than lower ranks (e.g. figure 2*b*, electronic supplementary material, figure S3b). The reason for the difference might be that our model implements specific mechanisms of hierarchy formation (reinforcement learning) and interference. Furthermore, compared to some reproductive-skew models, our model does not make use of concepts like concessions, negotiations or threats as possible explanations for sex differences in social dominance behaviour [49], but instead uses reinforcement learning as a mechanism that allows hierarchy formation. Our model, as well as our previous model [12], makes relatively detailed assumptions and is, therefore, more complex than previous reproductive skew models, but it can have the advantage of a somewhat closer match to field situations. This match might be further improved by incorporating more elements, such as age structure, relatedness between interacting group members, types of dispersal and explicit modelling of outside-option reproduction.

Game theory in biology started 50 years ago as an attempt to explain why animal contests are often settled without serious injury [16,51]. One important outcome [52] of this work is the idea that assessment of relative fighting ability permits settlement with little or no fighting, but this is unlikely to explain sex differences in the intensity of fighting. It follows that sex differences in fighting in social animals might be an important area where the general question of what limits aggression [16] can be explored, adding to the interest in the problem we study here. Our analysis suggests that the life-history context, including such things as the scale of competition, parenting consequences of contest damage, and uncontested reproductive opportunities, is a main factor explaining how costly contests become. A previous major result is that fighting evolves to become more costly if there are limited reproductive opportunities beyond the current contest [53] but, again, this is unlikely as a general explanation for sex differences in the intensity of fighting. Our work here thus adds a new perspective on the question of how costly aggression will be. A combination of ambitious empirical work, including comparative studies, and theoretical modelling might throw further light on the issue.

## Data Availability

C++ source code for the individual-based simulations is available at GitHub, together with instructions for compilation on a Linux operating system: https://github.com/oleimar/socdom4. Electronic supplementary material is available from Figshare [54].
